# F-box protein complex FBXL19 regulates TGFβ1-induced E-cadherin down-regulation by mediating Rac3 ubiquitination and degradation

**DOI:** 10.1186/1476-4598-13-76

**Published:** 2014-04-01

**Authors:** Su Dong, Jing Zhao, Jianxin Wei, Rachel K Bowser, Andrew Khoo, Zhonghui Liu, James D Luketich, Arjun Pennathur, Haichun Ma, Yutong Zhao

**Affiliations:** 1 Department of Immunology, Norman Bethune College of Medicine, Jilin University, Changchun, China; 2 Department of Anesthesia, First Hospital of Jilin University, Changchun, China; 3 Department of Medicine and the Acute Lung Injury Center of Excellence, University of Pittsburgh, 3459 Fifth Avenue, NW 628MUH, Pittsburgh 15213, PA, USA; 4 Department of Thoracic Surgery, University of Pittsburgh, Pittsburgh, PA, USA

**Keywords:** Small GTPase protein, Protein stability, E3 ligase, Ubiquitin-proteasome system, TGFβ1, E-cadherin

## Abstract

**Background:**

Rac3 is a small GTPase multifunctional protein that regulates cell adhesion, migration, and differentiation. It has been considered as an oncogene in breast cancer; however, its role in esophageal cancer and the regulation of its stability have not been studied. F-box proteins are major subunits within the Skp1-Cullin-1-F-box (SCF) E3 ubiquitin ligases that recognize particular substrates for ubiquitination and proteasomal degradation. Recently, we have shown that SCF^FBXL19^ targets Rac1 and RhoA, thus regulating Rac1 and RhoA ubiquitination and degradation. Here, we demonstrate the role of FBXL19 in the regulation of Rac3 site-specific ubiquitination and stability. Expression of TGFβ1 is associated with poor prognosis of esophageal cancer. TGFβ1 reduces tumor suppressor, E-cadherin, expression in various epithelial-derived cancers. Here we investigate the role of FBXL19-mediated Rac3 degradation in TGFβ1-induced E-cadherin down-regulation in esophageal cancer cells.

**Methods:**

FBXL19-regulated endogenous and over-expressed Rac3 stability were determined by immunoblotting and co-immunoprecipitation. Esophageal cancer cells (OE19 and OE33) were used to investigate TGFβ1-induced E-cadherin down-regulation by Immunoblotting and Immunostaining.

**Results:**

Overexpression of FBXL19 decreased endogenous and over-expressed Rac3 expression by interacting and polyubiquitinating Rac3, while down-regulation of FBXL19 suppressed Rac3 degradation. Lysine166 within Rac3 was identified as an ubiquitination acceptor site. The FBXL19 variant with truncation at the N-terminus resulted in an increase in Rac3 degradation; however, the FBXL19 variant with truncation at the C-terminus lost its ability to interact with Rac3 and ubiquitinate Rac3 protein. Further, we found that Rac3 plays a critical role in TGFβ1-induced E-cadherin down-regulation in esophageal cancer cells. Over-expression of FBXL19 attenuated TGFβ1-induced E-cadherin down-regulation and esophageal cancer cells elongation phenotype.

**Conclusions:**

Collectively these data unveil that FBXL19 functions as an antagonist of Rac3 by regulating its stability and regulates the TGFβ1-induced E-cadherin down-regulation. This study will provide a new potential therapeutic strategy to regulate TGFβ1 signaling, thus suppressing esophageal tumorigenesis.

## Background

Rac3 (ras-related C3 botulinum toxin substrate 3) is a member of the RhoGTPase family. In its active form, it is bound to GTP, whereas it is inactive in its GDP-bound form. It has been well studied that activation of RhoGTPases is controlled by guanidine activating proteins (GEFs) that exchange bound GDP to GTP and by GTPase activating proteins (GAPs) that promote GTP hydrolysis. Rac3 is enriched in the brains but it also expressed in a wide range of tissues [1]. Aberrant activation of Rac3 has been recognized to be important in tumor proliferation in both breast cancer and prostate cancer [2,3]. Rac3 regulates a variety of cellular functions including adhesion, cell migration, and differentiation. The controversial effects of Rac3 on cell migration have been reported. Rac3 negatively regulates diapedesis of prostate cancer cell PC3, since knockdown of Rac3 using Rac3 specific siRNA increased migration of PC3 cells through a bone marrow endothelial cell layer [4]. In contrast, overexpression of Rac3 promotes estrogen-induced breast cancer cell migration [2]. Rac3 was found to be involved in breast cancer cell aggressiveness through the activation of NF-κB and Erk2. Inhibition of Rac3 caused an increase in TNFα-induced apoptosis [5]. However, the role of Rac3 in the pathogeneses of esophageal cancer has not been studied.

Many posttranslational modifications, including ubiquitination, expand the size of the proteome exponentially and are pivotal in the regulation of protein stability [6-9]. The ubiquitin proteasome system (UPS) regulates protein ubiquitination, and therefore degradation and turnover, of many proteins vital of cellular regulation and function [10]. The UPS depends upon the action of three enzyme complexes. The E1 enzyme functions as an activator by creating a thioester bond between a cysteine of the E1 enzyme and the ubiquitin molecule via ATP hydrolysis. E2, known as the conjugating enzyme, then accepts the ubiquitin protein onto an active site cysteine. Finally, the E3 ligating enzyme complex is responsible for attachment of this ubiquitin protein to a lysine of the target protein [11]. Among the families of E3 ubiquitin ligases, the Skp1-Cullin-1-F-box protein (SCF) ligases complex is one of the largest. In this complex, the F-box protein is the substrate-recognition component [12,13]. F-box proteins have two main domains: an F-box motif and a substrate binding motif. F-box proteins use their F-box motif to bind to Skp1 and assemble the SCF ligase complex, whereas the substrate-binding motif is used for recognition and interaction with phosphorylated substrates [14]. Through an *in silico* search, the ‘orphan’ F-box protein FBXL19 has been identified and verified as an SCF E3 subunit [15]. Recently, we demonstrated that FBXL19 regulates interleukin (IL)-33 signaling by targeting its cognate receptor ST2L, for ubiquitination, which, in turn, triggers its proteasomal degradation to alter the innate immune response [16]. In addition to ST2L, we also found that Rac1 and RhoA are targets for FBXL19 and revealed new functions of FBXL19 in regulating cell migration, proliferation and cytoskeleton rearrangement [17,18].

E-cadherin, a type I classical cadherin, is a key component in the formation of cell-cell adherens-type junctions in epithelial tissues [19-21]. A variety of studies in cancers, including hepatocellular carcinoma, squamous cell carcinomas of the skin, head and neck, and pancreatic cancer, have demonstrated that E-cadherin plays a critical role as a tumor suppressor [22-25]. E-cadherin is often down-regulated during carcinoma progression and metastatic spread of tumors [26,27]. Loss of E-cadherin changes cancer cell phenotype and facilitates the initial invasive behaviors of epithelial-derived cancer [28]. Transforming growth factor β (TGFβ), a pleiotropic cytokine comprised of three isoforms in mammalian cells, function as a tumor promoting mediator in the later stages of cancers [29,30]. TGFβ1 signaling has been shown to play an important role in down-regulation of E-cadherin. It appears, that many epithelial tumors escape growth inhibition by TGFβ1, and TGFβ1 secretion by cancer may contribute to late tumor progression [31,32]. It has been shown that TGFβ1 expression is higher in esophageal cancer tissues, compared to normal squamous epithelium and non-malignant Barrett’s mucosa [33]. Over-expression of TGFβ1 in esophageal cancer is associated with advanced stage of disease and poor prognosis [34].

In this study, we demonstrate that Rac3 is a target protein of SCF^FBXL19^ E3 ligase. FBXL19 regulates Rac3 stability by ubiquitinating Rac3 on lysine 166 residue. This is the first report to reveal that Rac3 is implicated in TGFβ1-induced E-cadherin down-regulation in esophageal cancer cells. Further, we found that over-expression of FBXL19 attenuates the effect of TGFβ1 on E-cadherin down-regulation. This study will provide a molecular basis for SCF E3 ligase in the regulation of esophageal tumorigenesis.

## Results

### FBXL19 reduces Rac3 protein expression

**Figure 1:**
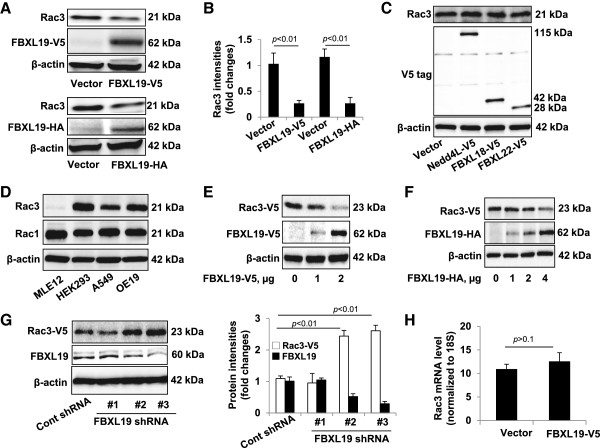
**FBXL19 induces Rac3 degradation - A.** HEK293 cells were transfected with V5 or HA tagged FBXL19 (FBXL19-V5 or FBXL19-HA) plasmid for 48 h. Cells lysates were analyzed by immunoblotting with Rac3, V5 tag, HA tag, and β-actin antibodies. **B**. Rac3 levels in Figure 1A were quantified by Image J software. **C**. HEK293 cells were transfected with V5 tagged Nedd4L, FBXL18, or FBXL22 plasmid for 48 h. Cell lysates were analyzed by immunoblotting with Rac3, V5 tag, and β-actin antibodies. **D**. Cell lysates from MLE12, HEK293, A549, and OE19 were analyzed by immunoblotting with Rac3, Rac1, and β-actin antibodies. **E**. MLE12 cells were co-transfected with V5 tagged Rac3 (Rac3-V5) and FBXL19-V5 (0–2 μg) plasmids for 48 h and then the cell lysates were analyzed by immunoblotting with V5 tag and β-actin antibodies. **F**. MLE12 cells were co-transfected with Rac3-V5 and FBXL19-HA (0–4 μg) plasmids for 48 h and then the cell lysates were analyzed by immunoblotting with V5 tag, HA tag and β-actin antibodies. **G**. MLE12 cells were co-transfected with Rac3-V5, Cont shRNA, or one of the three FBXL19shRNAs (#1, #2, and #3) plasmids for 48 h and then the cell lysates were analyzed by immunoblotting with V5 tag, FBXL19 and β-actin antibodies. Rac3-V5 levels were quantified by Image J software. **H**. Total RNA was extracted from empty vector- or FBXL19-V5 plasmid-transfected HEK293 cells, Rac3 mRNA levels were then examined by RT-real time PCR with specifically designed Rac3 primers. All the blots are representative of three independent experiments.

We have demonstrated that SCF^FBXL19^ ligase targeted Rac1 and RhoA for its ubiquitination and degradation [17,18]. To investigate if the FBXL19 also regulates Rac3 degradation, we transfected with V5-tagged FBXL19 (FBXL19-V5) and hemagglutinin-tagged FBXL19 (FBXL19-HA) plasmids in HEK293 cells respectively. Both forms of tagged-FBXL19 down-regulated endogenous Rac3 expression (up to ~73%) (Figure 1A-B), while over-expression of other E3 ligase subunits (NEDD4L, FBXL18, and FBXL22) had no effect on Rac3 expression (Figure 1C). To determine the specificity of the Rac3 antibody, we examined the Rac3 and Rac1 expression in several cell lines including MLE12, HEK293, A549, and OE19 cells. As shown in Figure 1D, Rac3 was highly expressed in HEK293 and OE19 cells compared to its expression in A549 cells. It was not detectable in MLE12 cells. Rac1 was highly expressed all the cell lines, suggesting Rac3 antibody does not cross-react with Rac1 protein. Further, we examined if FBXL19 reduces over-expressed Rac3 levels. Since Rac3 is not detectable in MLE12 cells (Figure 1D), we used MLE12 cells for studying the stability of over-expressed Rac3. We co-overexpressed V5-tagged Rac3 (Rac3-V5) with different doses of FBXL19-V5 or FBXL19-HA plasmid in MLE12 cells. Figure 1E and F show that the ectopic expression of FBXL19-V5 or FBXL19-HA diminished Rac3-V5 protein in a dose dependent manner. Further, we examined the effect of FBXL19 down-regulation on Rac3-V5 expression. MLE12 cells were transfected with Rac3-V5 and three individual FBXL19 shRNAs. As shown in Figure 1G, FBXL19 shRNAs (#2 and #3 only) reduced FBXL19 expression (up to ~50-70%), while Rac3-V5 protein mass was dramatically enhanced around 2.5 fold. To examine if the effect of FBXL19 on the reduction of Rac3 protein mass is due to modulation of Rac3 mRNA expression, we measured Rac3 mRNA levels by RT-realtime PCR. FBXL19-V5 had no effect on Rac3 mRNA expression, suggesting that FBXL19-mediated Rac3 reduction is through the regulation of Rac3 protein stability.

### FBXL19 induces Rac3 degradation in the proteasome system

**Figure 2:**
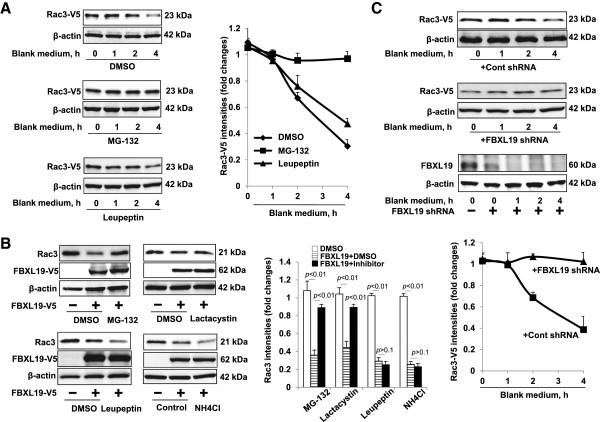
**FBXL19 induces Rac3 degradation in the proteasome system - A.** MLE12 cells were transfected with Rac3-V5 plasmid for 48 h. Cells were treated with MG-132 (20 μM) or leupeptin (100 μM) treatment for 1 h prior to change to blank medium for indicated incubation time. Cells lysates were analyzed by immunoblotting with V5 tag and β-actin antibodies. Rac3-V5 levels were quantified by Image J software. **B**. HEK293 cells were transfected with FBXL19-V5 plasmid for 48 h, and then the cells were treated with MG-132 (20 μM), Lactacystin (10 μM), leupeptin (100 μM), or NH4Cl (20 mM) for 4 h. Cells lysates were analyzed by immunoblotting with Rac3, V5 tag and β-actin antibodies. Rac3 levels were quantified by Image J software. **C**. MLE12 cells were co-transfected with Rac3-V5 and shFBXL19 plasmids for 48 h, and then the cells were cultured in blank medium (0–4 h). Cells lysates were analyzed by immunoblotting with V5 tag, FBXL19, and β-actin antibodies. Rac3-V5 levels were quantified by Image J software. All the blots are representative of three independent experiments.

In the process of investigating the degradation of the Rac3 protein, we examined if Rac3 degradation is mediated in the ubiquitin-proteasome system. We found that administration of FBS-free (blank) medium diminished Rac3-V5 in a time-dependent manner (Figure 2A). To identify which pathway is involved in the degradation of Rac3, Rac3-V5 overexpressed cells were treated with inhibitors of proteasomes (MG-132) or lysosomes (leupeptin) prior to administration of blank medium. Starvation-mediated Rac3 degradation was attenuated by pretreatment with MG-132 (up to ~67% at 4 h), but not leupeptin (Figure 2A), suggesting Rac3 degradation is mediated in the proteasome system, but not in the lysosome system. In addition, we found that the FBXL19-V5-induced Rac3 degradation was attenuated by MG-132 (up to 45-53%), but not by leupeptin (Figure 2B). The results were confirmed by treating cells with other inhibitors of the proteasome (Lactacystin) or lysosome (NH4Cl) (Figure 2B). To further investigate the role of FBXL19 in the regulation of Rac3 degradation, we down-regulated FBXL19 by FBXL19 shRNA transfection prior to treatment with blank medium. Figure 2C shows that FBXL19 shRNA transfection attenuated blank medium-mediated Rac3-V5 degradation (up to ~64% at 4 h), as well as FBXL19 expression (Figure 2C). The results suggest that FBXL19-mediated Rac3 degradation is in the proteasome system.

### FBXL19 targets Rac3 lysine^166^ for ubiquitination

**Figure 3:**
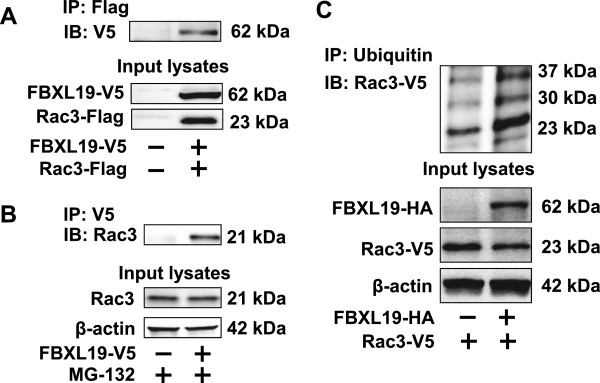
**FBXL19 targets Rac3 for ubiquitination - A.** HEK293 cells were co-transfected with Flag tagged Rac3 (Rac3-Flag) and FBXL19-V5 plasmids. Cell lysates were subjected to immunoprecipitation with a Flag tag antibody, followed by V5 tag immunoblotting. Input lysates were analyzed by immunoblotting with V5 tag and Flag tag antibodies. **B**. HEK293 cells were transfected with FBXL19-V5 plasmid followed by MG-132 (20 μM) treatment. Cell lysates were subjected to immunoprecipitation with a V5 tag antibody, followed by Rac3 immunoblotting. Input lysates were analyzed by immunoblotting with Rac3 and β-actin antibodies. **C**. MLE12 cells were co-transfected with Rac3-V5 and FBXL19-HA plasmids. Cell lysates were subjected to immunoprecipitation with an ubiquitin antibody, followed by V5 tag immunoblotting. Input lysates were analyzed by immunoblotting with HA tag, V5 tag and β-actin antibodies. All the blots are representative of three independent experiments.

**Figure 4:**
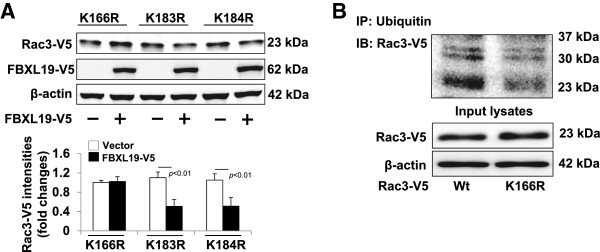
**Lysine 166 within Rac3 is an ubiquitin acceptor site for FBXL19 - A.** MLE12 cells were co-transfected with FBXL19-V5 wild type and Rac3 lysine mutant plasmids as indicated. Cell lysates were analyzed by immunoblotting with antibodies to V5 tag and β-actin. Rac3-V5 levels were quantified by Image J software. **B**. MLE12 cells were transfected with Wt Rac3-V5 or Rac3^K166R^-V5 plasmid. Cell lysates were subjected to immunoprecipitation with an ubiquitin antibody, followed by V5 tag immunoblotting. Input lysates were analyzed by immunoblotting with V5 tag and β-actin antibodies. All the blots are representative of three independent experiments.

To investigate if FBXL19 is associated with Rac3, we overexpressed V5-tagged FBXL19 and Flag-tagged Rac3 in HEK293 cells. Immunoprecipitation (IP) with an antibody to the Flag tag followed by Western blotting with an antibody to the V5 tag revealed that FBXL19-V5 associates with Rac3-Flag (Figure 3A). Further, endogenous Rac3 was detected in the FBXL19-V5 immunoprecipitation complex (Figure 3B). To examine if FBXL19 induces Rac3 ubiquitination, cells were transfected with Rac3-V5 and FBXL19-HA plasmids. Co-IP with an antibody to ubiquitin followed by Western blotting with V5 tag immunoblotting demonstrated that over-expression of FBXL19 increased polyubiquitination of Rac3 (Figure 3C). Lysine (K) residues within a target protein are ubiquitin acceptor sites for linking mono- or polyubiquitin [35]. To identify the putative ubiquitin acceptor site within Rac3 for FBXL19, we substituted several candidate K residues of Rac3 with arginine (R). Of several mutants tested, only Rac3^K166R^-V5, not Rac3^K183R^-V5 or Rac3^K184R^-V5, was resistant to FBXL19-mediated degradation (Figure 4A). Further, we compared the degree of ubiquitination of Rac3 wild type and Rac3^K166R^-V5 after incubation with MG-132. As shown in Figure 4B, polyubiquitinated Rac3 was immunoprecipitated by an antibody to ubiquitin, and Rac3 ^K166R^ exhibits less ubiquitination, compared to Rac3 wild type, suggesting that K166 is an ubiquitin acceptor site within Rac3.

### Role of N-terminus and C-terminus of FBXL19 in Rac3 degradation

**Figure 5:**
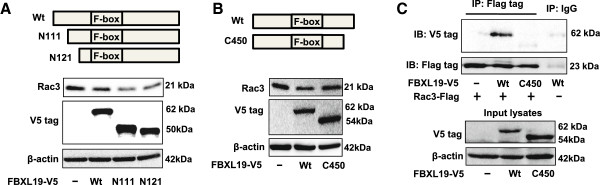
**Role of N-terminus and C-terminus of FBXL19 in Rac3 degradation - A.** HEK293 cells were transfected with plasmid encoding wild-type FBXL19-V5 (Wt) or an FBXL19 variant with truncation at the NH2 (N111 or N121). Cell lysates were analyzed by immunoblotting with Rac3, V5 tag, and β-actin antibodies. **B**. HEK293 cells were transfected with plasmid encoding wild-type FBXL19-V5 (Wt) or an FBXL19 variant with truncation at the COOH (C450). Cell lysates were analyzed by immunoblotting with Rac3, V5 tag and β-actin antibodies. **C**. HEK293 cells were co-transfected with Rac3-Flag and FBXL19-V5 wild-type (Wt) or V5 tagged FBXL19 variant with truncation at the COOH (C450) plasmids. Cell lysates were subjected to immunoprecipitation with a Flag tag antibody or IgG, followed by V5 tag immunoblotting. Input lysates were analyzed by immunoblotting with V5 tag, Flag tag and β-actin antibodies. All the blots are representative of three independent experiments.

Next we examined which part of FBXL19 is important in Rac3 degradation. Transfection of a plasmid encoding an FBXL19 variant with truncation at the NH2 (N111 or N121) resulted in increases in Rac3 degradation, compared to the wild type FBXL19-V5 (Figure 5A). This indicates that there is an inhibitory domain within the N-terminus of FBXL19. Further, an FBXL19 variant with truncation at the COOH (C450) lost the ability to degrade Rac3 (Figure 5B). In addition, co-IP with an antibody to the V5 tag revealed that wide type FBXL19-V5, but not the variant with truncation at the COOH (C450), associates with Rac3-Flag in HEK293 cells (Figure 5C). It suggests that Rac3 interacts with FBXL19 in the C-terminus of FBXL19.

### Rac3 regulates TGFβ1-induced E-cadherin down-regulation

**Figure 6:**
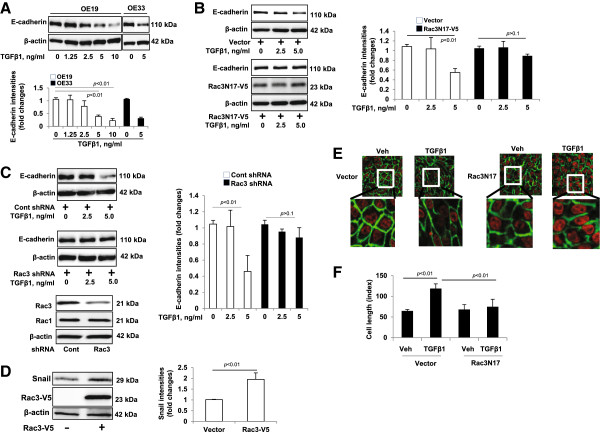
**Rac3 regulates TGFβ1-induced E-cadherin downregulation - A.** OE19 cells were treated with TGFβ1 (0–10.0 ng/ml) for 4 days or OE33 cells were treated with TGFβ1 (5 ng/ml) for 4 days, and then the cell lysates were analyzed by immunoblotting with E-cadherin and β-actin antibodies. E-cadherin levels were quantified by Image J software. **B**. OE19 cells were transfected with V5 tagged Rac3 inactive form (Rac3N17-V5) plasmid for 24 h followed by treatment of TGF-β1 (0–5 ng/ml) for 4 days. The cell lysates were analyzed by immunoblotting with E-cadherin, V5 tag, and β-actin antibodies. E-cadherin levels were quantified by Image J software. **C**. OE19 cells were transfected with Cont shRNA or Rac3 shRNA plasmid for 48 h followed by the treatment with TGF-β1 (0–5 ng/ml) for 4 days. The cell lysates were analyzed by immunoblotting with E-cadherin, Rac3, and β-actin antibodies. E-cadherin levels were quantified by Image J software. **D**. OE19 cells were transfected with Rac3-V5 plasmid for 48 h and then cell lysates were analyzed by immunoblotting with Snail, V5 tag, and β-actin antibodies. Snail levels were quantified by Image J software. **E**. OE19 cells were transfected with Rac3N17-V5 or vector plasmid for 24 h and then incubated with TGFβ1 (5 ng/ml) for 4 days. Cells were fixed and immunostained with E-cadherin (green). Nuclei were stained with DAPI (red). **F**. Cell length was measured by Image J software. All the blots and images are representative of three independent experiments.

TGFβ1 is associated with poor prognosis of esophageal cancer [34]. TGFβ1-mediated down-regulation of E-cadherin contributes to tumor invasion and proliferation [31]. Here, we show that TGFβ1 treatment (4 days) induces E-cadherin down-regulation in a dose dependent manner in esophageal cancer cell line OE19 (Figure 6A). Further, we found that TGFβ1 reduced E-cadherin expression in another esophageal cancer cell line OE33 (up to ~69%) (Figure 6A). To investigate if TGFβ1-mediated E-cadherin down-regulation is associated with Rac3, we overexpressed Rac3 inactive form (Rac3N17-V5) (Figure 6B) or down-regulation of Rac3 by transfection with Rac3 shRNA (Figure 6C) to inhibit Rac3 activity prior to TGFβ1 treatment (0, 2.5, 5.0 ng/ml, 4 days) in OE19 cells. Both the Rac3 inactive form and Rac3 shRNA attenuated the TGFβ1-reduced E-cadherin level (up to ~34-37%) (Figure 6B and C). Snail, a transcriptional factor, is well known to down-regulate E-cadherin. Here, we found that over-expression of Rac3-V5 increased Snail expression by ~1.95 fold in OE19 cells (Figure 6D). Immunostaining showed that TGFβ1 treatment induced OE19 cell elongation phenotype was attenuated by over-expression of Rac3N17 (Figure 6E and F). These results suggest that Rac3 is involved in TGFβ1-induced E-cadherin down-regulation.

### FBXL19 regulates TGFβ1-induced E-cadherin down-regulation

**Figure 7:**
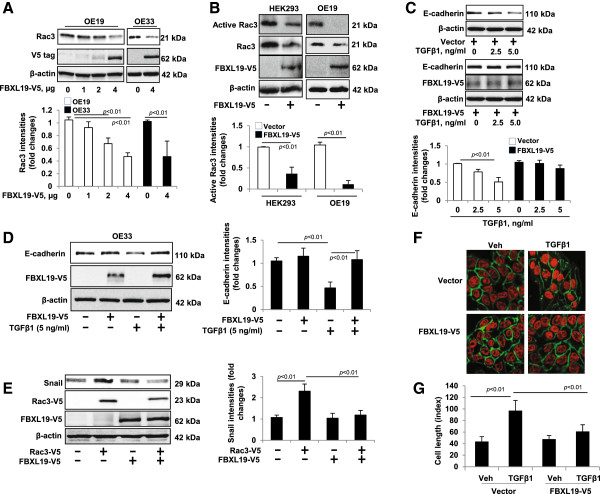
**FBXL19 regulates TGFβ1-induced E-cadherin down-regulation - A.** OE19 cells were transfected with FBXL19-V5 plasmid (0–4 μg) or OE33 cells were transfected with FBXL19-V5 (4 μg) for 2 days, and then the cell lysates were analyzed by immunoblotting with Rac3, V5 tag and β-actin antibodies. Rac3 levels were quantified by Image J software. **B**. Cell lysates from FBXL19-V5-overexpressed HEK293 or OE19 cells were analyzed for Rac3 activity by using a PBD-GTPγ-Rac3 pull down assay. Total cell lysates were analyzed by immunoblotting with Rac3, V5, and β-actin antibodies. Activated Rac3 levels were quantified by Image J software. **C**. OE19 cells were transfected with FBXL19-V5 plasmid for 24 h followed by treatment of TGFβ1 (0–5 ng/ml) for 4 days. The cell lysates were analyzed by immunoblotting with E-cadherin, V5 tag, and β-actin antibodies. E-cadherin levels were quantified by Image J software. **D**. OE33 cells were transfected with FBXL19-V5 plasmid for 24 h followed by treatment of TGFβ1 (5 ng/ml) for 4 days. The cell lysates were analyzed by immunoblotting with E-cadherin, V5 tag, and β-actin antibodies. E-cadherin levels were quantified by Image J software. **E**. OE19 cells were transfected with Rac3-V5, FBXL19-V5, or Rac3-V5 + FBXL19-V5 plasmids for 48 h. Cell lysates were then analyzed by immunoblotting with Snail, V5 tag, and β-actin antibodies. Snail levels were quantified by Image J software. **F**. OE19 cells were transfected with FBXL19-V5 or vector plasmid for 24 h and then incubated with TGFβ1 (5 ng/ml) for 4 days. Cells were fixed and immunostained with E-cadherin (green). Nuclei were stained with DAPI (red). **G**. Cell length was measured by Image J software. All the blots and images are representative of three independent experiments.

We have shown that FBXL19 mediates Rac3 degradation and Rac3 is involved in E-cadherin down-regulation by TGFβ1. Further, we examined if FBXL19 regulates TGFβ1-induced E-cadherin down-regulation. First, we confirmed the effect of FBXL19 in Rac3 degradation in OE19 and OE33 (up to ~53%) cells. As shown in Figure 7A, FBXL19-V5 diminished Rac3 protein in a dose dependent manner in OE19, as well as in OE33 cells. As expected, we found that FBXL19-V5 reduced Rac3 activity in both OE19 and HEK293 cells (up to 64-89%) (Figure 7B). Next, we overexpressed FBXL19-V5 in OE19 and OE33 cells for 24 h prior to TGFβ1 treatment (0, 2.5, 5.0 ng/ml, 4 days). Figure 7C shows that FBXL19-V5 attenuated the TGFβ1-induced decrease of E-cadherin (up to ~36% with TGFβ1 5 ng/ml treatment). These results were also observed in OE33 cells (up to ~62%) (Figure 7D). Further, we examined the effect of FBXL19-V5 on Rac3-mediated Snail expression. As shown in Figure 7E, FBXL19 blocked Rac3-V5 induced Snail expression (up to ~92% reduction) in OE19 cells. In addition, immunostaining showed that TGFβ1 treatment induced OE19 cell elongation phenotype was attenuated by over-expression of FBXL19 (Figure 7F and G) in OE19 cells. These results indicate that FBXL19 attenuates the effects of TGFβ1 on E-cadherin down-regulation and cell elongation phenotype by mediating Rac3 degradation.

## Discussion

Rho family GTPases are important intracellular signaling proteins that control diverse cellular functions, including actin cytoskeletal organization, migration and invasion, transcriptional regulation, cell cycle progression, apoptosis, vesicle trafficking, and cell-to-cell and cell-to-extracellular matrix adhesions [36,37]. Of the Rho family GTPases, Rac3 is implicated in regulating cell adhesion, growth, differentiation, and autophagy [38]. To date, little is known regarding the molecular regulation of Rac3 stability. Here, we show that Rac3 lifespan is regulated by the SCF^FBXL19^ E3 ligase and the proteasome system. FBXL19 targets Rac3 for ubiquitination in a specific lysine site, thus resulting in its degradation. Rac3, as an oncogene protein, plays a pivotal role in tumorgenesis of a variety type of cancers, including breast cancer and prostate cancer. This study is the first to report that Rac3 regulates TGFβ1-mediated E-cadherin down-regulation in esophageal cancer cells, indicating a critical role of Rac3 in the progress of esophageal cancer. Further, we demonstrate that FBXL19 negatively regulates Rac3-mediated TGFβ1 signaling in esophageal cancer. Here, we provide new evidence that ubiquitin E3 ligase contributes to the tumorgenesis of esophageal cancer. Targeting the ubiquitin E3 ligase will build a basis to develop a new potential therapeutic strategy to inhibit tumor growth and invasion. Despite the high homology in amino-acid sequence between Rac1 and Rac3, Rac3 differs from Rac1 in the COOH terminal region, which is involved in Rac localization and regulatory protein binding [1]. However, most of literature addressing the role of Rac in cancer progression concern Rac1, with little mention of Rac3. The elucidation of mechanisms for control of Rac3 protein stability therefore might have important implications for metastasis.

Post-translational modifications, including ubiquitination, regulate the function of key signaling proteins by modulating their activity, localization, and protein stability. Ubiquitination of small GTPases controls their behavior in cells, including migratory ability and cell cycle progression. We established recently that FBXL19 targets RhoA and Rac1 for ubiquitination and degradation, thus regulating cell growth, stress fiber formation, and cell migration [17,18]. F-box proteins have been shown to target multiple-substrates. For example, FBXW7 ubiquitinates multiple proteins involved in different signal pathways, such as Notch, cyclin, c-Myc and c-Jun, for ubiquination and degradation [39-41]. Here we uncover that Rac3 is a new substrate for FBXL19 and it interacts with C-terminus of FBXL19. This is the first study to investigate Rac3 ubiquitination and degradation. Over-expression of FBXL19 reduced Rac3 for disposal. In addition, we identified that FBXL19 induced Rac3 ubiquitination at lysine^166^. This acceptor site is similar to Rac1 ubiquitin acceptor site from FBXL19, which is distant from GTP binding site and resides within a C-terminal α-helix distinct from the polybasic tail [42]. It has been shown that other ubiquitin E3 ligases such as IAP and HACE1 also target Rac1 for ubiquitination and degradation. Since Rac3 shares a high homology with Rac1, the future study will focus on the role of IAP or HACE1 in regulation of Rac3 stability.

**Figure 8:**
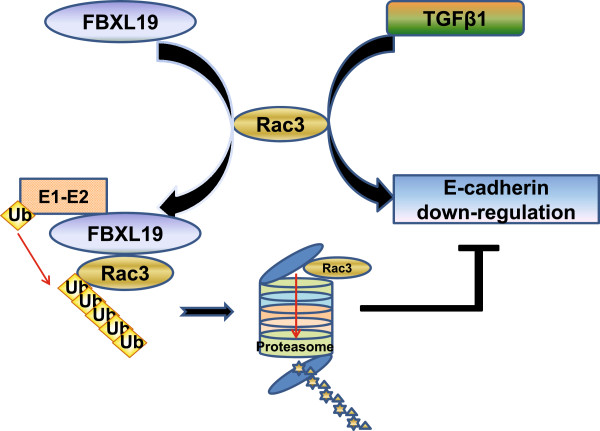
**FBXL19 regulates TGFβ1-induced E-cadherin down-regulation via targeting Rac3 for its degradation -** FBXL19 regulates Rac3 degradation in the proteasome system. TGFβ1 induces E-cadherin down-regulation. Over-expression of FBXL19 reduces Rac3 level, thereby inhibiting TGFβ1-induced E-cadherin down-regulation.

E-cadherin down-regulation is a fundamental biological process where epithelial cells lose their polarity and adopt morphology appropriate for migration [25]. Several studies have shown that E-cadherin down-regulation is important in cancer progression and is associated with poor outcome in several tumor sites, including non-small cell lung cancer, invasive ductal breast carcinoma, and gastric adenocarcinoma [43,44]. In tumors of the esophagus and gastroesophageal junction, disturbances in E-cadherin expression have been correlated with increasing invasive capacity, dedifferentiation, and lymph node metastases [45]. E-cadherin down-regulation occurs by canonical TGFβ1 signaling, which alters the function of the E-cadherin transcriptional repressor Snail directly. Rac1 has been known to regulate TGFβ1-mediated signaling; however, the role of Rac3 in TGFβ1-induced E-cadherin has not been studied. Rac3 is abundantly present in developing and adult brains, and mildly expressed in the heart, placenta and pancreas. Hajdo-Milasinovic, A. et al. demonstrated that Rac3 inhibited adhesion of neuronal cells [46], indicating Rac3 may regulate E-cadherin expression. In this study, we show that Rac3 is strongly expressed in esophageal carcinoma cells and it regulates Snail expression. Interestingly, inhibition of Rac3 by overexpressed inactive form of Rac3 (Rac3N17), Rac3 shRNA, or FBXL19 significantly attenuates TGFβ1-induced E-cadherin down-regulation (Figure 8). This study discovered that Rac3 plays an important role in the progress of esophageal cancer cells and the FBXL19 may function as a tumor suppressor through its ability to reduce Rac3 levels and inhibits TGFβ1 pathway. Future studies will be conducted to investigate the expression of Rac3 and FBXL19 in esophageal cancer tissues and adjacent normal tissues.

## Conclusions

In summary, the current study unveils Rac3 as a new molecular target of FBXL19. FBXL19 targets Rac3 for ubiquitination in lysine 166 and induces its proteasomal degradation. Inhibition of Rac3 by FBXL19 modulates E-cadherin expression in esophageal cancer cells. This is the first evidence to support that Rac3 plays a critical role in the tumorgenesis of esophageal cancer and that FBXL19 exhibits an anti-tumor property by down-regulation of small GTPase.

## Material and methods

### Cells and reagents

Esophageal adenocarcinoma (OE19 and OE33) cancer cells [Sigma-Aldrich (St. Louis, MO, USA)] were cultured with RPMI 1640 medium containing 2 mM glutamine and 10% FBS. HEK293 cells [Invitrogen (Carlsbad, CA, USA)] were cultured with DMEM medium containing 10% FBS. Murine lung epithelia (MLE12) cells [American Type Culture Collection (ATCC), Manassas, VA, USA] were cultured with HITES medium containing 10% FBS. All the cells were cultured at 37°C in 5% CO2. V5 antibody, E-cadherine antibody, mammalian expressional plasmid pcDNA3.1D/His V5 TOPO, *Escherichia coli* Top 10 competent cells, and recombinant TGFβ1 were from Invitrogen (Carlsbad, CA, USA). HA tag (29 F4), Flag tag (9A3), and ubiquitin (P4D1) antibodies were from Cell Signaling Technology (Danvers, MA, USA). Rac3 antibody was from Proteintech Group (Chicago, IL, USA). FBXL19 antibody was from Abgent (San Diego, CA, USA). Leupeptin, ammonium chloride (NH4Cl), β-actin antibody, individual FBXL19 shRNAs, and scrambled shRNA were from Sigma Aldrich (St. Louis, MO, USA). MG132 and lactacystin from Calbiochem (La Jolla, CA, USA). Immunobilized protein A/G beads were from Santa Cruz Biotechnology (Santa Cruz, CA, USA). Superfect transfection reagent was from QIAGEN (Valencia, CA, USA). All materials in highest grades uses in the experiments are commercially available.

### Construction of FBXL19 and Rac3 plasmids

The FBXL19 cDNA was inserted into a pcDNA3.1D/V5-His vector (Invitrogen, CA, USA). Site directed mutagenesis was performed to generate Rac3 lysine or serine mutant according to the manufacturer’s instructions (Agilent Technologies, Santa Clara, CA, USA).

### Immunoblotting and immunoprecipitation

Cells were washed with cold PBS and collected in cell lysis buffer containing 20 mM Tris HCl (pH 7.4), 150 mM NaCl, 2 mM EGTA, 5 mM β glycerophosphate, 1 mM MgCl2, 1% Trison X100, 1 mM sodium orthovanadate, 10 μg/ml protease inhibitors, 1 μg/ml leupeptin, and 1 μg/ml pepstatin. An equal amount of cell lysates (20 μg) was subjected to SDS-PAGE, electrotransferred to membranes and immunoblotted as described previously [17,18]. For immunoprecipitation, equal amounts of cell lysates (1 mg) were incubated with specific primary antibodies overnight at 4°C, followed by the addition of 40 μl of protein A/G agarose and incubation for additional 2 h at 4°C. The immunoprecipitated complex was washed 3 times with 1% Triton X100 in ice cold phosphate-buffered saline and analyzed by immunoblotting with indicated antibodies.

### Immunostaining

OE19 cells were plated on 35 mm glass-bottom culture dishes. After treatment, cells were fixed in 3.7% formaldehyde for 20 min, followed by permeabilization with 0.1% Triton X100 for 2 min. Cells were incubated with 1:200 dilution of E-cadherin, followed by a 1:200 dilution of fluorescence-conjugated secondary antibody sequentially for immunostaining. Immunofluorescent cell imaging was performed on a Nikon confocal microscope.

### Plasmid transfection

HEK293 cells and OE19 cells were subcultured on 6-well plates or 35 mm plates for 24 h. Superfect transfection reagent was used for HEK293 cells and lipofectamine transfection reagent was used for OE19 cells. The transfection reagent was added to the mixture of 3 μg of plasmid and 200 μl of reduced serum medium, mixed, and incubated for 10 minutes to allow transfection reagent/DNA complexes to form. And then the mixture was added directly to the cells. The transfected cells were cultured for 48 h. shRNA plasmids were also delivered into cells with the same protocol and the cells were cultured for 72 h.

### Rac3 activity assay

FBXL19-V5 transfected HEK293 and OE19 cells were cultured in 100-mm dishes and Rac3 activation was evaluated using a PBD-GTPγ-Rac3 pull down assay. Briefly, cell lysates (0.5-1 mg/ml) were loaded with 10 μg of PAK-1 p21-binding domain fusion-protein conjugated to agarose for 1 h to bind Rac3-GTP, centrifuged, and washed three times with lysis buffer. The proteins were separated by SDS-PAGE, transferred to nitrocellulose membranes, and probed with a Rac3 antibody. Total cell lysates were also probed separately with anti-Rac3 and anti-β-actin antibodies to confirm equal loading.

### TGFβ1 treatment

Esophageal cancer cells were cultured in 6-well plates or 35 mm dishes in 10% FBS medium. After 48 h of transfection, the medium was replaced with 1 ml of serum-free (blank) medium. TGFβ1 was added into the medium with the concentration range of 0, 1.25, 2.5, 5.0 10 ng/ml. After 4 days of incubation at 37°C, the cells were collected and analyzed by immunoblotting with E-cadherin antibodies.

### Statistics

All results were subjected to statistical analysis using two-way analysis of variance, and, wherever appropriate, analyzed by Student-Newman-Keuls test. Data are expressed as mean ± SD of triplicate samples from at least three independent experiments and values that were p < 0.01 were considered statistically significant.

## Abbreviations

Rac3: Ras-related C3 botulinum toxin substrate 3; UPS: Ubiquitin proteasome system; SCF: Skp1-Cullin-1-F-box protein; IL-33: Interleukin 33; GEFs: Guanidine activating proteins; GAPs: GTPase activating proteins; TGFβ: Transforming growth factor β; co-IP: Co-immunoprecipitation.

## Competing interests

The authors declare that they have no competing interest.

## Authors’ contributions

SD performed the most experiments and prepared the manuscript; JZ, JW, RKM, AK, YZ assisted Western blotting and immunostaining; ZL, HM, JDL, AP, YZ provided financial support to SD and provided discussion; AP, RKM, YZ edit the manuscript; YZ, JZ, SD designed the experiments. All authors read and approved the final manuscript.

## Authors’ information

YZ, HM, and ZL are the corresponding authors of this work.
